# Type 3 innate lymphoid cells dominate the ILC compartment in endstage lung disease

**DOI:** 10.3389/fimmu.2026.1716115

**Published:** 2026-06-10

**Authors:** Olga Halle, Jan-Niklas Falke, Claudia Kessemeier, Khatuna Lobjanidze, Emily Fuchshuber, Sean Brüske, Svenja Gaedcke, Marina Schumacher, Londa Dähne, Jonas Knaup, Maresa Borghorst, Sophia Pallenberg, Melanie Albrecht, Adan Chari Jirmo, Jana Bergmann, Michelle Paulsen, Danny Jonigk, Peter Braubach, Anna-Maria Dittrich

**Affiliations:** 1 Department for Pediatric Pneumology, Allergology and Neonatology, Hannover Medical School, Hannover, Germany; 2 German Center for Lung Research, Biomedical Research in Endstage and Obstructive Lung Disease (BREATH), Hannover, Germany; 3 Department of Respiratory Medicine and Infectious Diseases, Hannover Medical School, Hannover, Germany; 4 Department of Translational Pulmonology, University of Heidelberg, Heidelberg, Germany, and Translational Lung Research Center Heidelberg (TLRC), German Center for Lung Research (DZL), Heidelberg, Germany; 5 Institute of Pathology, University Medical Center, RWTH University Aachen, Aachen, Germany; 6 Department for Pathology, Hannover Medical School, Hannover, Germany; 7 Cluster of Excellence RESIST (EXC 2155), German Research Foundation (DFG), Hannover Medical School, Hannover, Germany

**Keywords:** CFTR modulator therapy, chronic inflammatory disease, cystic fibrosis, elexacaftor/tezacaftor/ivacaftor (ETI), emphysema, endstage lung disease, idiopathic pulmonary fibrosis, innate lymphoid cells (ILC)

## Abstract

Mucosal innate lymphoid cells (ILCs) act as cytokine producers in first line defense but also as contributors to chronic inflammation. We have previously shown that ILCs belong to those non-conventional lymphocytes promoting an IL-17A-rich tissue environment in endstage lung tissue. Here, we provide an in-depth characterization of ILCs in lung and lung-draining lymph nodes (LNs) from patients with three endstage lung diseases, i.e. cystic fibrosis (CF), chronic obstructive pulmonary disease (COPD)/emphysema, and pulmonary fibrosis, which reveals critical differences to healthy lung tissue. Our analyses show that type 3 ILCs dominate the ILC compartment in lungs and LNs from these three endstage lung disease entities, where they contribute to the pro-inflammatory cytokine milieu in the tissue, whereas type 1 ILCs constitute the major ILC population in healthy lung tissue. In contrast to the endstage situation, in the peripheral blood (PB) of clinically stable CF patients, we find type 2 ILCs at increased frequencies compared to healthy controls. In CF patients receiving the CFTR modulator elexacaftor/tezacaftor/ivacaftor (ETI), these differences in PB ILC composition are sustained for up to 24 months, in spite of significant reductions of systemic inflammation, which accompany strong improvements in lung function. These findings suggest that the local and systemic ILCs compartments reflect unique immunological aspects of chronic lung disease which appear challenging to address by disease-modifying treatment.

## Introduction

Innate lymphoid cells (ILCs) lack cognate antigen receptors but produce cytokines in response to signals from the tissue environment. Like activated CD4^+^ T cells, fully differentiated ILCs are subdivided into distinct sets of “helper ILCs”, i.e. ILC1s, ILC2s, and ILC3s, that produce cytokines supporting type 1, 2, or 3 immunity (1, 2). Type 3 ILCs, through production of IL-17 family cytokines, contribute to chronic inflammation and tissue remodeling (3). We have recently shown ILCs producing IL-17A to be detected in a murine model of cystic fibrosis (CF)-like lung disease (4) and in lung and LN tissues from patients with endstage CF (4, 5) as well as COPD/emphysema (EM), and pulmonary fibrosis (FI) (5). Along with other non-conventional lymphocytes, ILCs appear to play a role in chronic inflammatory processes and in driving perpetual inflammation (3, 6). However, their distribution and role in the pathogenesis of endstage lung disease remains to be elucidated (7).

Here, in order to study the contribution of ILCs to endstage lung disease in-depth, we examined lung and lung-draining lymph node (LN) tissues explanted from patients with CF, EM, and FI at the time of lung transplantation and compared the ILC compartment in these tissues to healthy lung tissue and lymph nodes. Moreover, we analyzed the ILC compartment in peripheral blood (PB) samples from clinically stable CF patients enrolled in the MODULATE-CF trial (NCT04732910) at Hannover Medical School and undergoing triple CFTR modulator therapy with ETI. We report that pro-inflammatory ILC3s dominate the ILC compartment in endstage lungs and LNs, and we gauge the impact of ILC-derived cytokines on the tissue environment. In the PB of MODULATE-CF participants compared to healthy blood donors, we find type 2 ILCs to be increased in frequency, an alteration that persists even after 24 months of inflammation-reducing CFTR modulator therapy and may thus reflect hard-wired pathological processes, which are challenging to address even with effective disease-modifying treatment.

## Materials and methods

### Patient characteristics

Lung and/or LN tissue from a total of 58 lung donors and 107 lung transplant recipients was analyzed in this study (**Supplementary Table 1**). Samples were obtained between December 2017 and January 2025 from n=22, n=37, and n=28 explanted lungs, and from n=20, n=41, and n=27 LNs of patients with cystic fibrosis (CF), COPD/emphysema (EM) or lung fibrosis (FI), respectively, undergoing lung transplantation at Hannover Medical School. Lung donor (LD) lung tissue was obtained from downsizing and unilateral transplantation surgeries in n=9 cases. LD LNs from lung transplant donors (n=51) were resected from the trachea as part of the transplantation procedure.

PB samples were obtained from 48 participants enrolled in the MODULATE-CF trial at Hannover Medical School (8–13) before and at 3, 12, and 24 months post onset of treatment with the CFTR modulator elexacaftor/tezacaftor/ivacaftor (ETI) (**Supplementary Table 2**). No acute exacerbations were present at the visits when the samples were taken. 16 healthy blood donors (HD) were recruited at Hannover Medical School via a public announcement on the basis of being non-smokers, not receiving immunosuppressive or anti-inflammatory medication and not suffering from any chronic inflammatory condition (**Supplementary Table 2**).

Acquisition of all samples and tissue specimen was approved by the Hannover Medical School ethics committee (votes 10748-_BO_S_2023, 101989_BO_K_2023, 2700‐2015) and performed following informed consent of patients or their legal guardians. Lung function measurements (ppFEV_1_) of the MODULATE-CF cohort, outside of their association with ILC composition, have already been published elsewhere (8–13).

### Cell isolation from human tissues

Lung and LN samples were kept in RPMI medium supplemented with 2.5% FCS at 4 °C until processing. Lung tissue was disrupted with a gentleMACS dissociator (Miltenyi, Bergisch Gladbach, Germany) and the resulting pieces were incubated in digestion buffer (RPMI, 2.5% FCS, 2 mg/mL Collagenase Type III, Worthington (Worthington Biochemical Corporation, Lakewood, NJ, USA), 10 μg/mL DNase I (Sigma‐Aldrich, Taufkirchen, Germany)) at 37 °C for 35 min. Digestion was stopped by adding EDTA to a final concentration of 0.01 mM, followed by centrifugation and replacement of digestion buffer. LN tissue was mechanically disrupted. Single‐cell suspensions were generated by filtering lung tissue digests and LN pieces consecutively through a metal mesh and cell strainers (100 μm, Greiner Bio‐One, Kremsmünster, Austria), followed by red blood cell lysis. PBMCs were obtained from EDTA blood tubes using density gradient centrifugation. To this end, EDTA blood was diluted 1:2 in PBS, added on top of a layer of BioColl separating solution (density 1.077 g/mL) and centrifuged for 20 min at 460g and 20 °C. The resulting interphase was harvested, washed with PBS containing 2.5% FCS, and processed for flow cytometric staining.

### Flow cytometric analysis

For flow cytometric staining, cell suspensions were incubated with Fc blocking reagent (human TruStain FcX; BioLegend) and incubated with antibodies against cell surface antigens, followed by viability stain (Pacific Orange; Thermo Fisher), and intracellular staining using the True Nuclear Transcription factor Buffer set (BioLegend, 424401) throughout. For cytokine staining, cell suspensions were adjusted to a concentration of 1 × 10^7^ cells per mL and incubated with PMA (0.1 μg/mL; Sigma‐Aldrich) and ionomycin (0.75 μg/mL; Sigma‐Aldrich) for 4 h in the presence of GolgiPlug Protein Transport Inhibitor (BD Biosciences) before blocking and staining. Antibodies were: FITC-labelled CD3 (UCHT1), CD4 (RPA-T4), CD11c (Bu15), CD19 (HIB19), CD14 (HCD14), CD34 (561), CD94 (DX22), CRTH2-PE (BM16), CD117-PerCP-Cy5.5 (A3C6E2), CD127-PE-Cy7 (A019D5), CD45-APC (HI30), CD161-BV421 (NKR-P1A), and NKp44-biotin (P44-8) with streptavidin-APC-Cy7 reagent (BioLegend) for surface staining and anti-human IFN-γ (4S.B3), IL-22 (IL22JOP), IL-17A (BL168), IL-5 (TRFK5) for intracellular staining. Data were acquired using a FACS Canto II (BD Biosciences) and analysed using FlowJo software (version 10; Treestar), employing the gating strategy described in **Supplementary Figure 1** and the Supplementary Methods section. To assess cytokine production in ILC subsets, a minimum threshold of 20 cells was set for the count of cells in a specific subpopulation as a criterium for further analysis. In addition to the 20 cell-threshold, flow cytometric analyses in which a high (> 1%) proportion of a specific ILC subset produced a particular cytokine were assessed through a second quality check. To avoid false positive findings datapoints were manually excluded if the proportion of cells producing the cytokine was based on only very few cells analyzed or on cells with a very low cytokine signal. While most subpopulations in LN samples contained many more cells, giving us reliable information on cytokine production, owing to the small number of cells obtained, the same analysis was not feasible for lung ILC subpopulations and for ckit^+^ ILC2s in LNs.

### Histological analysis

**Figure 1:**
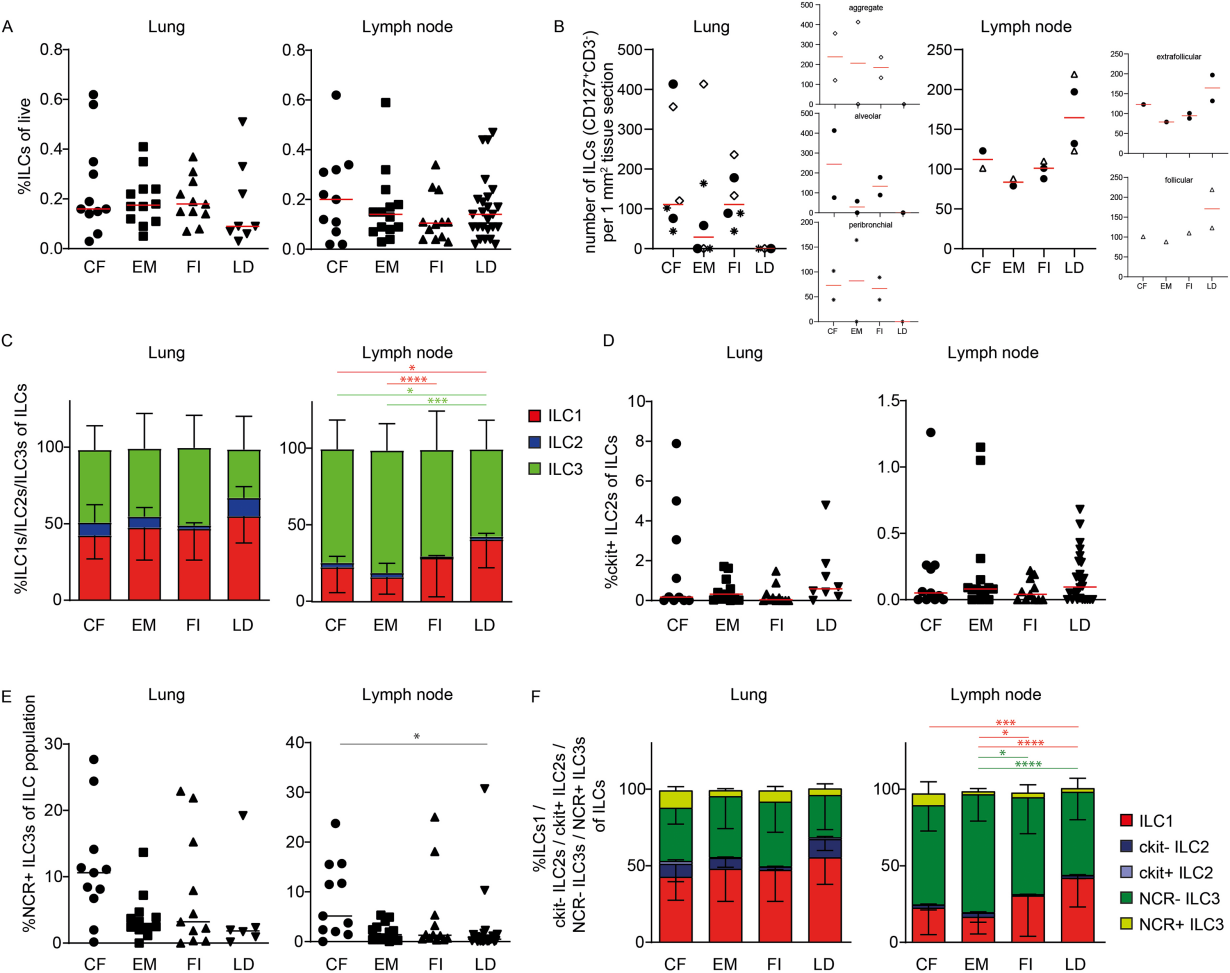
ILCs accumulate in endstage lungs with ILC3 as a dominant population. **(A)** Frequencies of ILCs (Lineage^-^CD127^+^ cells, see **Supplementary Figure 1**) among live cells in endstage lungs (left panel) and lymph nodes (right panel) from patients with endstage cystic fibrosis (CF), emphysema (EM), pulmonary fibrosis (FI), and from healthy lung donors (LD) as determined by flow cytometry. Horizontal red lines indicate median; one symbol per patient (n=11/12/11/8 patients for lungs and n=11/14/12/25 patients for LNs from CF/EM/FI/LD, respectively). Red numbers indicate fold median change compared to LD. Kruskal-Wallis test overall p-value =0.7042. **(B)** Frequencies of ILCs (CD127^+^CD3-) as determined using immunofluorescence microscopy of lung and LN tissue from CF, EM, FI patients and LDs. Lungs from n=2 patients (CF, EM, FI) and n=1 patient (LD) as well as LNs from n=1 (CF, EM) and n=2 patients (FI, LD) are shown. Counting was performed in defined anatomical regions marked as follows: alveolar region (•), lymphocyte aggregate (◊), peribronchial region (*) for lungs, and follicular (Δ) and extra-follicular region (•) for LNs. Symbols represent means of 3–6 technical replicates per patient and region. Red lines indicate median. No inferential statistics was performed because of the small number of patients available for analysis. **(C)** ILC subset composition in endstage lungs (left panel) and lymph nodes (right panel) from patients with CF, EM, FI and LDs as determined by flow cytometry. Percentage of ILC1s, ILC2s, ILC3s among all ILCs (Lineage-CD127^+^). Bars and whiskers show mean+SD of individual patients (n=11/12/11/8 for lungs and n=11/14/12/14 for LNs from CF/EM/FI/LD, respectively). Two-way ANOVA with Tukey’s multiple comparisons test; asterisks refer to pairwise comparisons between indicated disease entities and color-coded ILC subsets. Non-indicated pairwise comparsions are n.s. **(D)** Proportion of ckit^+^CRTH2^+^ ILCs among ILCs in lung and lymph nodes of indicated patient groups. Horizontal lines indicate median of patients (n=10/11/11/8 patients for lungs and n=11/14/11/26 patients for LNs from CF/EM/FI/LD, respectively). Kruskal-Wallis with Dunn’s multiple comparison test. **(E)** Proportion of NCR^+^ ILC3s among ILCs in lung and lymph nodes of indicated patient groups. Horizontal lines indicate median of patients (n=11/12/11/6 patients for lungs and n=11/14/12/23 patients for LNs from CF/EM/FI/LD, respectively). Kruskal-Wallis with Dunn’s multiple comparison test. **(F)** Overall composition of ILCs in lung and LN including ckit^+^CRTH2^+^ ILCs (i.e. ckit^+^ ILC2s) and NCR^+^ ILC3s. Please note that ckit^+^ ILC2s are not included in the ILC2 subset depicted in **(C)**, whereas NCR^+^ ILC3s, together with NCR^-^ ILC3s, make up the ILC3 population depicted in **(C)**. Mean+SD of patients (n=11/12/11/8 patients for lungs and n=11/14/12/14 patients for LNs from CF/EM/FI/LD, respectively). Two-way ANOVA with Tukey’s multiple comparisons test; asterisks refer to pairwise comparisons between indicated disease entities and color-coded ILC subsets. Non-indicated pairwise comparsions are n.s.

Immunofluorescence staining was performed as described in (5). Antibodies were anti‐CD3 (mouse anti‐human CD3; BioLegend 300440) and anti‐CD127 (mouse anti‐human CD127; Beckman, IM1980U) antibodies. Representative images with a qualitative analysis of this data set have been published previously in (5). Here, for quantification, regions of interest (ROIs) were defined in anatomically distinct regions of the images, i.e. alveolar region, lymphocyte aggregates, and peribronchial regions for lungs, and follicular and extrafollicular regions for LNs. Per patient and anatomical region, ILCs were enumerated in 3–6 ROIs sized 150µm x 150 µm and numbers were averaged from all ROIs per patient and anatomical region. Numbers were then normalized to a denominator of 1 mm^2^ to obtain the number depicted in the **Figure 1B**. Note that the limited number of tissues available for this type of analysis precluded performance of inferential statistical analysis on the data set. Sample sizes are given in the figure caption.

### ChipCytometry analysis

Single cell suspensions obtained from human lung and LN tissues were enriched using STEMCELL technologies EasySep human ILC enrichment Kit (cat no. 17975) according to the manufacturer’s instructions. Enrichment efficacy was tested using flow cytometric staining with anti-human CD127 antibody and anti-Lineage (Lin) cocktail as described above. In the ILC-enriched fraction, 34.2% to 81.7% of cells were CD127-positive and 34.8% to 96.7% of cells were Lineage-negative. The ILC-enriched fraction was mounted onto a ChipCytometry chip (Canopy Biosciences, formerly Zellkraftwerk GmbH, Hannover, Germany (14, 15),) and consecutive stainings were performed with pre-established combinations of the following anti-human antibodies: PerCP-Cy5.5-labelled CD127 (A019D5), CD294 (BM16), CD161 (HP-3G10), CD152 (BNI3), FITC-labelled CD94 (DX22), CD279 (MIH4), IL-23R (218213), ICOS (ISA-3), CD3 (UCHT1), CD4 (RPA-T4), CD11c (Bu15), CD19 (HIB19), CD14 (HCD14), CD34 (561), PE-labelled CD117 (104D2), NKp44 (P44-8), CD196 (11A9), CD154 (TRAP1), RANKL (MIH24), B7-H2 (2D3/B7-H2), TRAIL (RIK-2), BAFF (137314), CD40 (82111), CD80 (2D10), CD45 (HI30). Following every staining round, images were obtained using a Zeiss Axio Imager M2 automated microscope equipped with a Plan Apochromat ×20/0.8 objective and fitted with a Basler scA1400-17gm monochromatic camera, followed by a bleaching step. Following bleaching, background images were obtained for control purposes. In total, one or two chips were analyzed per disease entity and organ. For image analysis, in every fluorescence channel, background signal was subtracted from the fluorescence signal detected following staining. Image registration of the consecutively acquired images of the same regions was performed using ZellExplorer application (Zellkraftwerk), with automated cell segmentation followed by manual inspection and, if required, addition of non-detected or deletion of incorrectly identified cells. Overexposed, incorrectly focused or images with artefacts (bubbles, fibres, crystals etc.) were excluded from analysis. CD45^+^ cells were selected based on the histogram depiction of fluorescence intensities in stained vs. background images. Fluorescence expression values of CD45^+^ cells were exported for all markers. Data sets were enriched for ILCs by selecting the respective proportion of Lineage^-^CD127^+^ cells identified in the flow cytometric enrichment control. For display in heatmaps, expression of the ILC population was averaged across every chip. Note that the limited number of tissues available for this type of analysis precluded performance of inferential statistical analysis on the data set. Sample sizes are given in the figure caption.

### Bioinformatic analysis

Cluster analysis of ILCs was performed on expression values obtained from analyzing the ChipCytometry. To this end, on the basis of the enrichment efficacy as determined by flow cytometric analysis (see above), the respective fractions of CD127^+^ and Lin^-^ cells were selected from the list of all cells acquired on the chip. For cluster analysis the data was first logarithmic transformed. The data was then split by organ and clustered using Leiden algorithm (leidenalg version 0.8.10, number of neighbors = 15, resolution= 1). For each cluster, the number of cells per disease was determined and the mean values (based on raw data) for each of the markers were calculated for each disease/cluster combination.

The used script can be found under https://github.com/sgaedcke/ChipCytometry_ILCs_endstage.

### Statistical analysis

Statistical significance was calculated using GraphPad Prism version 9 and 10 for Windows. Where non-parametric tests were available for the experiment design in question, we used those for analysis. For two-factor designs, a parametric test was used to accommodate the need to handle missing values in the data set rather than using imputation. Missing values were considered to be missing completely at random, e.g. because of typical obstacles in the daily clinical routine. For statistical tests, a P‐value limit of P < 0.05 was accepted to indicate statistical significance. In figures, asterisks indicate P < 0.05 (*), P < 0.01 (**) and P < 0.001 (***), and P > 0.05 (n.s., not significant), respectively. The respective statistical tests applied are named in the figure legends.

## Results

### ILCs accumulate in endstage lungs with ILC3 as a dominant population

To characterize the ILC compartment in endstage lungs, we analyzed cells obtained from lung and LN tissue explanted at the time of lung transplantation and compared these to donor lung tissue and LNs from lung donors using flow cytometry. In lung tissues from patients with endstage CF, EM, and FI, we found the median percentage of ILCs among live cells to be elevated 1.8-fold, 1.9-fold, and 2.0-fold, respectively, as compared to LD (median 0.16% in CF, 0.18% in EM, 0.18% in FI vs. 0.09% in LD; **Figure 1A**, left panel), indicating an accumulation of ILCs in endstage lung tissue. Overall, these data point towards an increase in the number of ILCs in endstage lung tissue.

Complementing the flow cytometric approach, we used lung and LN tissues available from 1–2 patients per disease entity to quantify ILCs using immunofluorescence microscopy (**Figure 1B**; representative images with a qualitative analysis have been published in (5)). While owing to the low sample availability, generalized conclusions from the histological analysis were not feasible, in the comparison of the available lung tissue from CF and FI compared to LD, we detected increased median numbers of ILCs, identified as CD127^+^CD3- cells (**Figure 1B**, left panels). In lung-draining LNs analyzed by flow cytometry, the median percentage of ILCs among live cells was higher in CF than in EM than in FI, with LD at about the same median percentage as EM (**Figure 1A**, right panel). Consistently, no clear disease entity-specific differences were detectable in LNs using immunofluorescence microscopy analysis (**Figure 1B**, right panels).

To determine which ILC subtypes were present in endstage lung tissue, we further subdivided total ILCs into type 1, 2, and 3 ILCs (ILC1s, ILC2s, ILC3s) using the surface markers CD117 (ckit) and CD294 (CRTH2) (**Supplementary Figure 1A** and (16)). Of note, NK cells were largely excluded from the total ILC population using NK cell marker CD94 in the lineage mix, so total ILCs referred to in the subsequent analyses encompass the helper ILC populations only. In healthy lung donor tissue (LD), ILC1s (ckit-CRTH2- ILCs) accounted for the largest proportion among total ILCs, i.e. median 57.7% (IQR 44.8%-70.0%), whereas ILC2s (ckit-CRTH2^+^ ILCs) made up the smallest portion, i.e. median 11.2% (IQR 5.4%-19.4%), and ILC3s (ckit^+^CRTH2- ILCs) amounted to median 24.6% (IQR 16.8%-49%) of the total ILC population (**Figure 1C**, left panel). This composition was different in lung tissue from all three endstage lung disease entities. The median proportion of ILC1s among total ILCs was 38.3% in CF, 53.6% in EM, and 51.8% in FI, respectively. Concomitantly, the proportion of ILC2s among total ILCs was even lower in endstage lungs than in LD lungs, accounting for only 2.4%, 5.8%, and 2.5% of ILCs in CF, EM, and FI, respectively. Intriguingly, the percentage of ILC3s among total ILCs was substantially increased in all three endstage lung diseases compared to LD lungs, reaching medians of 46.1% in CF, 39.9% in EM, and 46.6% in FI, respectively (**Figure 1C**, left panel).

Compared to lung tissue, in LNs from all groups analyzed, ILC1s accounted for a smaller median proportion of total ILCs than in lungs (**Figure 1C**, right panel). Also, ILC2s were detectable at even smaller median frequencies in LNs than lungs from any of the groups, including LDs. As in lungs, ILC3s dominated the ILC compartment in LNs from all three endstage diseases, accounting for a median of 74.8% (CF), 86.9% (EM), and 80.3% (FI) of total ILCs as compared to 57.3% in LD LNs (**Figure 1C**, right panel). In CF LNs, the decrease in the ILC1 and increase in ILC3 subpopulations compared to LD LNs was statistically significant, as was the increase of ILC3s in EM LNs compared to LD LNs (**Figure 1C**, right panel). Altogether, these changes indicate a shift in the ILC composition in endstage lung and lung-draining LN tissue towards an ILC3-dominated population, primarily at the expense of the ILC1 population.

Consecutively, we analyzed the helper ILC compartment in further detail by including ckit-expressing ILC2s (17, 18) and subdividing the ILC3 population based on the additional surface marker NKp44 (19). In both lung and LN tissues, we were able to detect a small population of ILCs co-expressing ckit and CRTH2 (**Figure 1D**; **Supplementary Figure 1A**), which have been categorized previously as an ILC2 population with the potential to produce type 3 cytokines (17, 18). Altogether, ckit^+^CRTH2^+^ ILCs were exceedingly rare, accounting for a median 0.17%, 0.32%, 0.04%, and 0.59% of ILCs in lung tissues from CF, EM, FI, and LD, respectively (**Figure 1D**, left panel). In LNs, the percentage of ckit^+^CRTH2^+^ cells among ILCs ranged between 0.04% (FI) and 0.10% (LD) of ILCs (**Figure 1D**, right panel). No disease entity-specific differences were observed in the ckit^+^CRTH2^+^ population in either lung or LNs. Subdivision of the ILC3 population based on the expression of natural cytotoxicity receptor (NCR) NKp44, which marks activated ILC3s ( (19), **Supplementary Figure 1A**) revealed fewer NCR^+^ ILC3s than NCR- ILC3s in all organs and entities. The median frequency of NCR^+^ ILC3s among ILCs was highest in CF lung (10.6%) and CF LN (5.2%), with a significantly higher proportion in CF LN than LD LN (**Figure 1E**). In other organs and entities, the median proportion of NCR^+^ ILC3s among ILCs was 0.5%-3.2% (**Figure 1E**). An overall composition of the ILC compartment including ckit^+^ ILC2s and subdivision of ILC3s on the basis of NKp44 is shown in **Figure 1F**. Altogether, these analyses suggest that in endstage lung tissues, and most pronounced in CF, the most strongly IL-17A-driven of these endstage disease entities (5), the composition of the ILC compartment changes in favor of an ILC3-dominated population.

### ILC3 dominance is associated with an IL17A-rich environment, with over-proportional contribution of ILCs to IL-17A production

**Figure 2:**
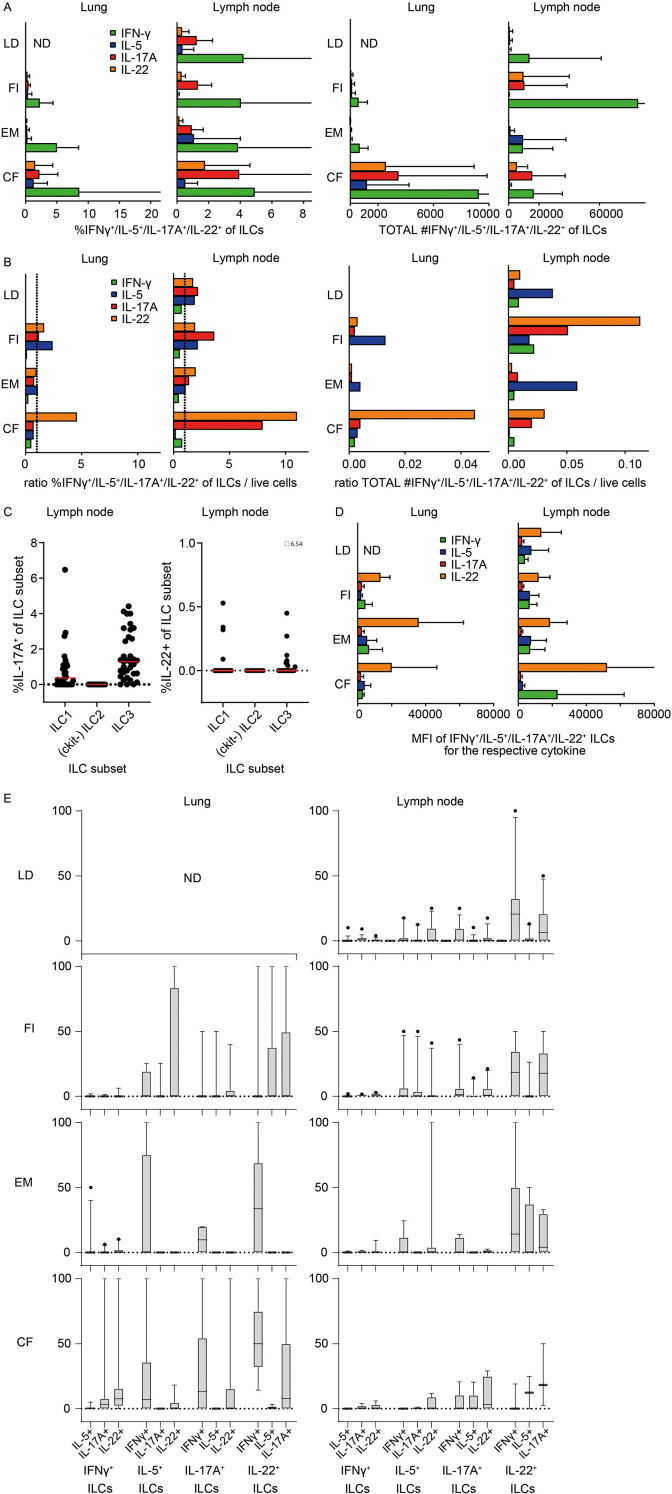
ILC3 dominance is associated with an IL17-rich environment, but IL-17 is provided by several ILC subtypes. **(A)** Stimulus-evoked expression capacity of cytokines IFN-γ, IL-5, IL-17, and IL-22 in ILCs from lung and lymph nodes from lung donors **(LD)** or patients with endstage cystic fibrosis (CF), emphysema (EM), pulmonary fibrosis **(FI)** as determined by flow cytometry following incubation with PMA/ionomycin for 4 hrs (n=8/11/12/0 patients for lungs and n=7/9/10/16 patients for LNs from CF/EM/FI/LD, respectively). Measurements are given as percentage of cytokine-expressing cells among ILCs (left set of panels) and total number (#) of cytokine-positive ILCs (right set of panels). Bars indicate mean+SD of the percentage of cytokine-positive cells among ILCs (Lin^-^CD127^+^). Note that cytokine measurements were not performed on LD lungs due to the limited availability of these tissues (ND, no data). **(B)** Contribution of ILC cytokines to live cell cytokine production. Mean percentage of cytokine-positive cells among ILCs (left set of panels) or mean total number of cytokine-positive ILCs (right set of panels) was divided by mean percentage of cytokine-positive cells among live cells (left) or mean total number of cytokine-positive live cells (right) to calculate the depicted ratio, respectively. n=8/11/12/0 patients for lung and n=7/9/10/16 patients for LN from CF/EM/FI/LD, respectively. **(C)** IL-17 and IL-22 production in ILC1, (ckit-) ILC2, and ILC3 subpopulations in LNs from n=26 patients (16 EM, 2 FI, 8 LD). Please note that LNs from CF patients were not available for the analysis shown in **(C)** One symbol per patient, horizontal lines indicate median. Lung ILC subpopulations and LN ckit^+^ ILC2s frequently comprised fewer than 20 cells and were excluded entirely from analysis. Please refer to materials and methods for further information on quality control and exclusion criteria. **(D)** MFI of ILCs expressing IFN-γ, IL-5, IL-17A, or IL-22 in the respective flow cytometric analysis channel. Bars indicate mean+SD of n=5-7/3-10/8-10/0 patients for lung and n=3-5/6-9/10-11/10–14 patients for LN from CF/EM/FI/LD, respectively, with ranges of n determined by which cytokine was analyzed. Note that absolute comparison of MFIs between different cytokines is not informative. **(E)** Co-expression analysis of cytokines in ILCs. Diagrams show the percentage of ILCs positive for the cytokines IFN-γ, IL-5, IL-17A, or IL-22 that also express a second cytokine simultaneously. Bars+whiskers show 10^th^ to 90^th^ percentile; individual dots represent data points outside this range; horizontal lines in boxes represent median. n= 8/11/9/0 patients for lung and n=5/9/10/16 patients for LN from CF/EM/FI/LD, respectively.

We next analyzed PMA/ionomycin stimulus-invoked production capacity for the cytokines IFN-γ, IL-5, IL-17A, and IL-22 in endstage lung and LN ILCs (**Figure 2A**, left set of panels). Detection of lung ILCs producing IL-17A and IL-22 was most prominent in CF compared to EM and FI, although IL-17A-producing ILCs were detected in LNs from all groups analyzed. In contrast, IL-22 production in LN ILCs was most pronounced in CF (**Figure 2A**, left set of panels). IFN-γ was the cytokine produced by the largest fraction of ILCs in lungs and LN from all disease entities (**Figure 2A**, left set of panels). In comparison, IL-5 production was negligible in all tissues and disease entities (**Figure 2A**, left set of panels). Also when analyzing the total number of cytokine-producing ILCs, CF was the disease entity with the most prominent population of ILCs producing IL-17 and IL-22 in the lung, while in LN, ILCs producing IL-17 and IL-22 were detectable in both CF and FI (**Figure 2A**, right set of panels).

Since many other cells besides ILCs produce cytokines in chronically inflamed tissues, we sought to understand whether ILCs would contribute to a specific polarization of the immune response more than other cell types by calculating the ratio of the percentage of cytokine-producing ILCs to the percentage of live cells producing the same cytokine (**Figure 2B**, left set of panels). The proportion of IFN-γ^+^ cells within the ILC population was lower than within the live cell population in both lungs and LNs from all disease entities (**Figure 2B**, left set of panels, green bars), suggesting that although ILCs produce IFN-γ, production of this cytokine is not their distinguishing feature. Instead, the cytokines that were produced to a greater degree by ILCs than other live cells were IL-17A and IL-22, particularly in CF lung and LNs (**Figure 2B**, left set of panels, orange bars). In CF, IL-22 production was 4.6-fold (lung) and 11.0-fold (LN) higher in ILCs than in live cells. IL-17A production in LNs was 7.9, 1.4, 3.6., and 2.2-fold higher among ILCs in LNs than among live cells in LNs (**Figure 2B**, left set of panels, red bars). Altogether, these data suggest that ILCs contribute over-proportionally to the production of IL-17 family cytokines and that accordingly, this contribution is particularly pronounced in the most IL-17A-driven of the diseases analyzed, CF. Because ILCs are a numerically very small population, we sought to estimate their contribution to overall cytokine production by calculating also the total ratio of cytokine-producing ILCs to cytokine-producing live cells (**Figure 2B**, right set of panels). As would be expected, this ratio was substantially below 1.0 for all entities and cytokines (**Figure 2B**, right set of panels), however, the strongest contribution of ILCs to IL-17 and IL-22 production was still detected in CF lungs as well as CF and FI LNs (**Figure 2B**, right set of panels).

To understand if production of IL-17 family cytokines within the ILC population originated exclusively from ILC3s, we co-stained IL-17A, IL-22 and the surface markers required to distinguish the ILC subtypes (**Figure 2C**). To overcome the challenge of low total cell and thus low ILC numbers in the tissues analyzed, data from different disease entities were pooled in this analysis. Notably, through the concomitant effects of the Sars-CoV2 pandemic and the availability of the new CFTR triple modulator therapies ETI, lung transplantations for CF patients ceased almost completely at our center (20), preventing inclusion of meaningful data for this disease entity. Furthermore, cell counts for ILCs in the lung were too low to perform subpopulation-specific analysis for cytokine production, as were cell counts for IL-22-producing ILCs in the LNs (see Materials and Methods). However, subdivision of IL-17A^+^ LN ILCs into ILC1s, ckit- ILC2s, and ILC3s, which was feasible with respect to cell numbers, revealed that IL-17A was produced mainly, but not exclusively, by ILC3s, with IL-17A^+^ cells accounting for 1.4% of ILC3s and 0.2% of ILC1s in LNs (**Figure 2C**, left panel). With the given set of tissue samples not including CF LNs, IL-22 expression was very low in all subsets, with the positive cells detected in the ILC3 and ILC1 fraction (**Figure 2C**, right panel). Altogether, these results show that endstage lungs exhibit a strongly pro-inflammatory, type 3-skewed environment with a varying degree of IL-17A cytokine production by ILCs and, at least in LNs from EM and FI patients, both type 1 and type 3 ILCs contributing to IL-17A production.

To aim at understanding if the contribution of ILCs to the pro-inflammatory IL-17A-rich environment in endstage lungs was driven primarily by changes in the ILC composition or alternatively by increases in per-cell output, we also analyzed the MFI of ILC-expressing cytokines in lungs and LNs. This analysis did not reveal substantial differences between the disease entities (**Figure 2D**). Furthermore, we sought to understand if ILCs in lungs and LNs produced several of the measured cytokines simultaneously (**Figure 2E**). Considering the four cytokines analyzed in this study, the fraction of cytokine-expressing ILCs that co-expressed a second one of the cytokines analyzed was typically very low. Expression of a second cytokine in IFN-γ-positive ILCs was detected almost exclusively in CF lungs, with typically fewer than 25% of IFN-γ-positive ILCs producing a second cytokine. Larger percentages of cytokine-positive ILCs producing a second cytokine were found in particular among IL-22-positive ILCs in LNs, albeit this was the case in LD LNs as well as LNs from endstage lungs (**Figure 2E**). Interpretation of these analyses requires strong caution because of the small number of cytokine-producing ILCs in the analysis and needs to consider the nature of the experiment setup, i.e. analyzing PMA/ionomycin-stimulus-induced cytokine production. Nevertheless, the results suggest that increases in ILC numbers contribute more strongly to enhancing the pro-inflammatory environment in endstage lungs than an increase in per-cell production of pro-inflammatory cytokines in ILCs and that the described change in ILC composition may thus be an central contributor.

### ILCs in endstage lung diseases show distinct proinflammatory expression profiles

**Figure 3:**
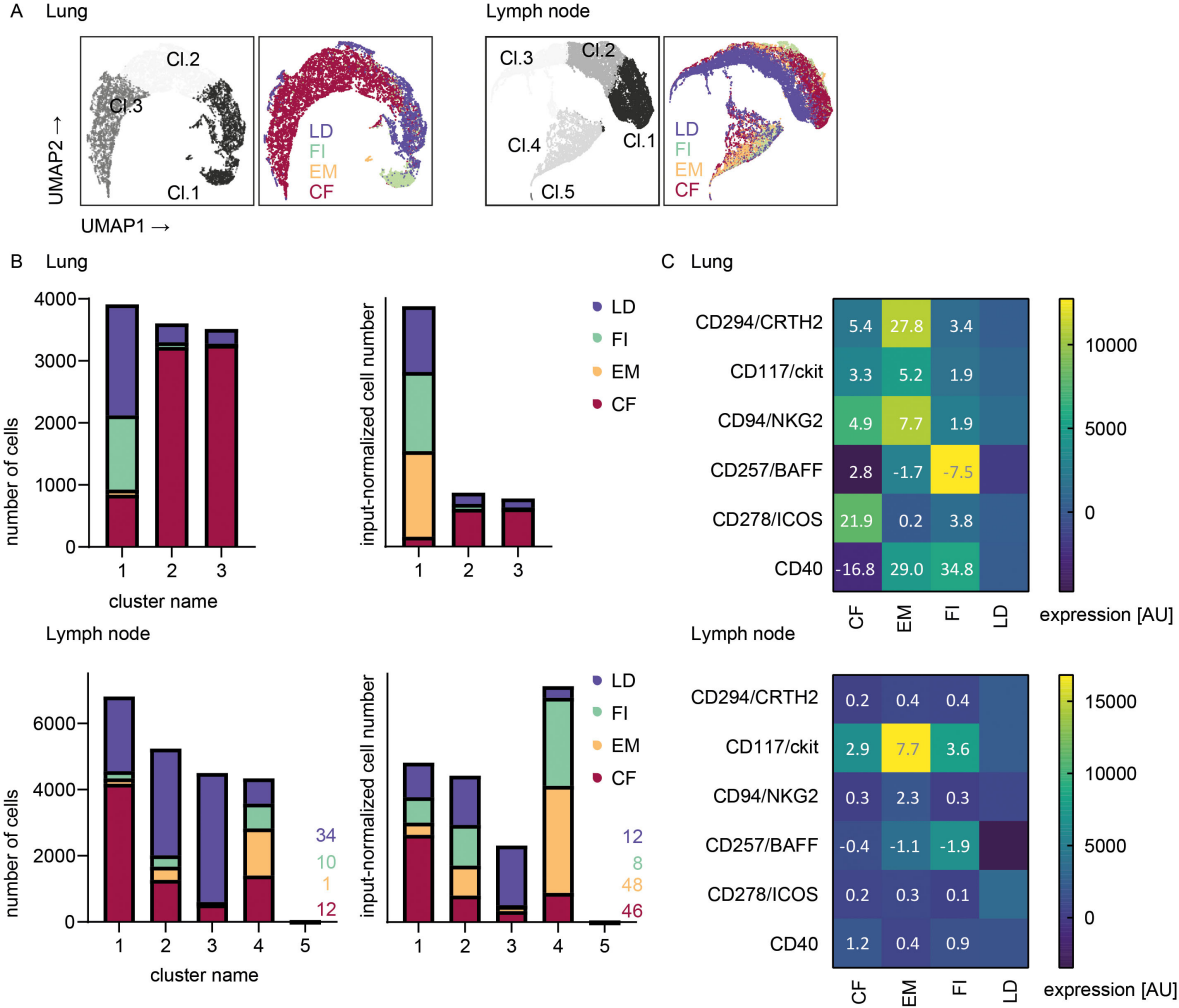
ILCs show disease-specific expression pattern **(A)** ILC-enriched cell suspensions from lung and LN of patients with endstage CF/EM/FI and from LD were analyzed using chip cytometry. CD127^+^Lineage^-^ cells were enriched from microscope images and mean expression per chip was analyzed for every marker. Cluster analysis was performed using a selected set of “identity markers”, i.e. CD45, Lineage mix, CD127/IL7Rα, CD294/CRTH2, CD117/ckit, and CD94/NKG2. Greyscale plots (left) show allocation of cells to clusters as indicated; color-coded plots (right) show representation of cells from the different diseases in the respective clusters. **(B)** Left-hand plots: Allocation of cells from different disease entities to individual clusters as visualized in color-coded plots in **(A)** Please note that the numbers of cells that were included in the analysis differs between disease entities. Right-hand plots: same data following input-normalization to simulate homogenous representation of diseases in input cell population. For lymph nodes, cluster 5, color-coded numbers indicate number of cells in respective cluster. **(C)** Expression of selected markers on bioinformatically enriched CD127^+^Lineage^-^ cells. Color-code indicates mean expression of all cells on the chip (or average if 2 chips were available). Numbers indicate fold mean expression compared to LD. Only markers which were changed at least 2-fold in CF vs. LD are displayed. A-C. For lung, n=1/1/2/1 patients (1 chip/patient), and for LN, n=1/2/2/1 patients (1 chip/patient) analyzed from CF/EM/FI/LD, respectively.

To deepen our characterization of the ILC compartment in the individual disease entities, in addition to the flow cytometric and histological characterization, a small set of available tissues were analyzed using chip cytometry, a microscopic analysis technique using iterative staining of cells fixed in a fluid-filled chamber on a microscope slide (14, 15). Prior to their application onto the chip, ILC populations were enriched from cell suspensions obtained from endstage lung and LN tissues. Based on expression of CD127 and the Lin mix, we selected those cells on the chip qualifying as *bona fide* ILCs based on the flow cytometric enrichment analysis and then performed cluster analyses based on a selected subset of markers, i.e. CD127, CD94, CD117, CD294, Lin, CD45. Cluster analysis resulted in the formation of 3 distinct clusters in lung and 5 distinct clusters in LN tissue (**Figure 3A**). The representation of cells from the different disease entities within individual clusters varied between clusters, with cells from all entities represented in comparatively similar proportions in lung cluster 1 and LN cluster 4, whereas in lung, clusters 2 and 3 were dominated by cells from CF, and in LNs, cluster 3 comprised predominantly cells from LD (**Figure 3B**, left plots). For input-normalized analyses, the number of cells from a given disease present in an individual cluster was adjusted to simulate an even representation of all diseases in the input cell population (**Figure 3B**, right plots). These input-normalized analyses confirmed our previous observations, showing that cells from CF dominated lung clusters 2 and 3, and cells from LD dominated LN cluster 3.

To characterize the dominant disease-specific populations in the analyzed patients more detail, we determined the mean expression of all markers on the *in silico*-enriched ILCs (**Figure 3C**). Consistent with the enrichment of ILC3 subpopulations in endstage lungs and LNs, CD117/ckit-expression was elevated in lung and LN ILCs of all three disease entities compared to LD ILCs (**Figure 3C**). In CF lung ILCs, the most prominent differences in expression compared to LD lung ILCs were found for CD294/CRTH2, CD94/NKG2, and CD278/ICOS, which were 5.4, 4.9, and 21.9-fold higher, respectively, than in LD lung ILCs, and for CD40, which was 16.8-fold lower than in LD lung ILCs (**Figure 3C**). In EM lung ILCs, the markers with the most prominent difference compared to LD lung ILCs were CD294/CRTH2 and CD40 (27.8 and 29.0-fold increase, **Figure 3C**). In FI lung ILCs, CD40 expression was 34.8-higher than in LD lung ILCs (**Figure 3C**). In LN ILCs, expression differences were less prominent than in lung; however, consistent with the ILC3 dominance observed in the flow cytometric analysis, CD117/ckit expression in CF, EM, and FI LN ILCs was elevated 2.9, 7.7, and 3.6-fold compared to LD LN ILCs (**Figure 3C**). The above-described results must be interpreted with caution because of the restricted availability of the tissues for analysis and the correspondingly low sample size (see figure caption for details). Yet, they may point to the existence of distinct inflammatory expression profiles in ILCs from endstage lungs and LNs and also indicate changes in the polarization of the immune response in the tissue as indicated by the changes in cytokine production reported above (**Figures 2A–C**).

### Clinically stable CF patients show an altered ILC compartment in the PB which does not normalize within 24 months of CFTR modulator triple therapy ETI

**Figure 4:**
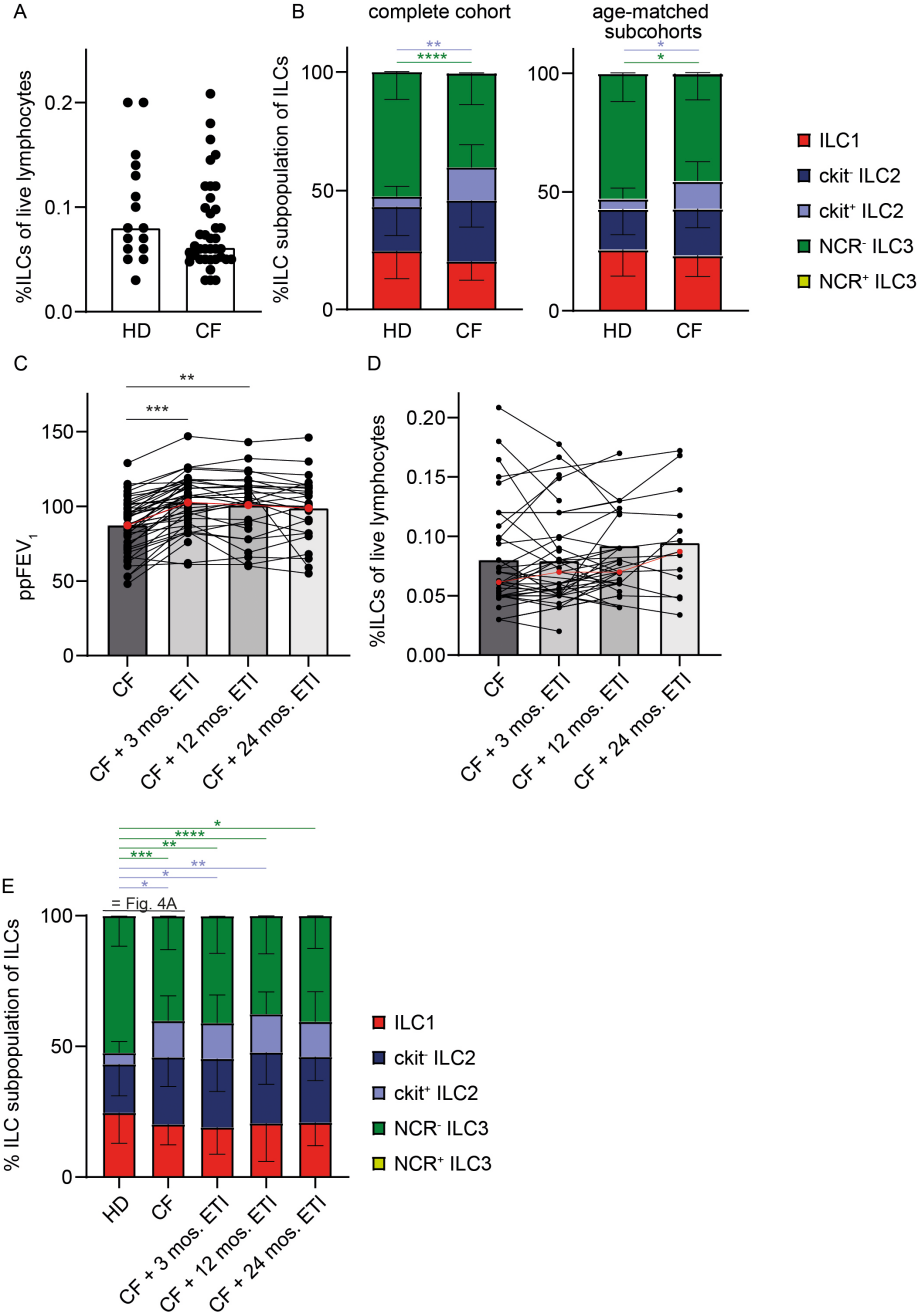
The ILC compartment in PB of CF patients is characterized by an increase in ILC2s at the expense of ILC1s and the major NCR- ILC3 population but remains stable under ETI treatment **(A)** Percentage of ILCs among live lymphocytes in PB of n=16 healthy donors (HD) and n=38 MODULATE-CF participants at baseline (CF). Bars represent median, one symbol per patient, Mann-Whitney test. **(B)** Left panel: ILC composition in PB of n=16 HDs and n=38 MODULATE-CF participants at baseline (CF) (left panels). Right panel: ILC composition in age-matched subcohorts (n=15 HDs and n=10 MODULATE-CF participants; please refer to Supplementary Materials for details). Mean+SD of subjects, two-way ANOVA and Tukey’s multiple comparisons test; asterisks refer to pairwise comparisons between indicated disease entities and color-coded ILC subsets. Non-indicated pairwise comparsions are n.s. **(C)** ppFEV_1_ of patients in the MODULATE-CF cohort (CF) examined 3, 12, and 24 months after onset of treatment. Note that ppFEV_1_ values are shown as an indicator of clinical improvement and have previously been published elsewhere (see Materials and methods). One set of line+dots per patient; red line+dots indicate mean of patients (n=46/47/30/26 patients at baseline/3/12/24 months, respectively). Kruskal-Wallis test with Dunn’s multiple comparisons test. **(D)** Percentage of ILCs among live lymphocytes throughout the course of ETI treatment. Bars indicate mean, red set of line+dots indicates median of patients (n=38/39/33/13 patients at baseline/3/12/24 months, respectively). ANOVA with Tukey’s multiple comparisons test. **(E)** ILC composition in PB of MODULATE-CF participants throughout the observation period compared to HDs. Note that groups HD and baseline data **(B)** are shown again to simplify the comparison. Mean+SD of patients (n=16/28/39/33/13 donors or patients for HD/baseline/3/12/24 months, respectively). Two-way ANOVA with Tukey’s multiple comparisons test; asterisks refer to pairwise comparisons between indicated disease entities and color-coded ILC subsets. Non-indicated pairwise comparsions are n.s.

With ILC3s being the dominant ILC population in lungs from patients with endstage CF, EM, and FI, we tested whether a similarly altered composition could be detected systemically, i.e. in the peripheral blood, at an earlier stage in the disease process and would be amenable to disease-modifying interventions. To this end, we chose to analyze PB mononuclear cells (PBMC) samples from patients enrolled into the MHH arm of the MODULATE-CF cohort receiving the CFTR triple modulator therapy elexacaftor/tezacaftor/ivacaftor (ETI) (see Materials and methods) as a causal, disease-modifying treatment approach and compared these to PBMCs from HDs (**Supplementary Table 1**). As opposed to the lung and LN donors undergoing lung transplantation, MODULATE-CF participants were clinically stable, with ppFEV_1_ values of mean ± SD 87.25 ± 17.55 at the baseline visit, which – as expected – increased significantly following the onset of therapy (see below and **Figure 4E**).

Regarding the frequency of total ILCs in the PB, no statistically significant difference was detected between HDs and MODULATE-CF participants at the baseline visit (**Figure 4A**). Analyzing the ILC composition in the PB of HDs, NCR- ILC3s were the most frequent ILC subpopulation, accounting for mean 52.4% of ILCs, followed by ILC1s (24.7% of ILCs), ckit- ILC2s (18.6% of ILCs) and ckit^+^ ILC2s (4.3% of ILCs) (**Figure 4B**, left panel). NCR^+^ ILC3s were virtually absent from HD blood (0.07% of ILCs) (**Figure 4B**, left panel). In the PB of MODULATE-CF participants at the baseline visit (CF), NCR- ILC3s were still the most frequent subpopulation, accounting for the majority (39.5% ± 13.1%) of ILCs, but their number was significantly reduced compared to HDs (**Figure 4B**, left panel). Concomitantly, ILC2s in MODULATE-CF participants were more frequent than in HDs, with ckit- ILC2s elevated to a mean proportion of 25.7% among ILCs, and ckit^+^ ILC2s making up a significantly higher proportion among total ILCs than in CF (13.9%, **Figure 4B**, left panel). Mean ILC1 frequencies in CF PB were lower than in HD (**Figure 4B**, left panel). Overall, the comparison between PBMCs from HD und CF patients thus indicated that although NCR^-^ ILC3s remain the most frequent ILC subset in the PB also in CF patients, ILC2s are more frequent in the PB of CF patients than HD, at the expense of the ILC1 and ILC3 populations. To address the possibility that differences in the ILC composition between the HD and the MODULATE-CF cohort may be due to age differences between the cohorts, we selected age-matched sub-cohorts as described in Supplementary Materials and performed the same analysis. Although this approach reduced the sample size available for analyses, ILC composition as well as the main differences between HD and MODULATE-CF cohort remained stably visible also in the age-matched sub-cohort analysis (**Figure 4B**, right panel).

To further understand the alterations of the ILC compartment in the PB of CF patients, we followed the ILC subset composition in MODULATE-CF participants for the course of 24 months post initiation of ETI treatment. Within that time frame, consistent with previous publications (21), we observed drastic improvements in clinical presentation including ppFEV_1_, which increased from mean ± SD 87.25 ± 17.55 (baseline) to 102.6 ± 16.50 (3 months visit) and remained stable thereafter (100.8 ± 20.3 at 12 months, and 98.85 ± 21.36 at 24 months, **Figure 4C**, data also included in (8)). However, throughout the entire time, the percentage of ILC among live lymphocytes (**Figure 4D**) and the ILC subset composition in the PB remained entirely stable (**Figure 4E**). The mean proportion of NCR- ILC3s was still significantly smaller in MODULATE-CF participants at the 24-month-visit compared to HDs and the mean proportion of ckit^+^ ILC2s continued to be significantly larger than in HDs until the 12-months-visit with the given sample size (**Figure 4E**). Altogether, these observations suggest that alterations in the PB ILC composition in CF remain present long after clinical presentation and lung function have improved through the initiation of disease-modifying therapy.

## Discussion

Progressive tissue damage in endstage lung disease is driven at least in part by IL-17 family cytokines (6), with contributions from T_H_17 cells as well as γδ-T cells, CD8^+^ T cells, NK T cells and ILCs (5). Here, we gauge the contribution of ILCs to chronic lung inflammation in endstage lung disease. Using flow cytometric and chip cytometric analysis, we show that ILCs accumulate in endstage lungs, with type 3 ILCs constituting the dominant ILC population in lungs and lung-draining LNs obtained from patients with these diseases. Comparing patients with CF, lung fibrosis, and COPD/lung emphysema as the etiologies underlying the necessity for lung transplantation in our cohort, we did not observe overt differences regarding the ILC composition. These data point to a uniform endstage pathway characterized by increased contributions of ILC3s and their associated cytokines to lung failure.

Previous reports support the idea that sustained changes in the ILC tissue composition may be a hallmark of chronic lung inflammation leading to progressive lung failure. Specifically, in COPD lungs, the percentage of ILC1s among ILCs was reported to be increased compared to control patients with no COPD, with no overt corresponding changes in ILC2 or ILC3 populations, while the total ILC number remained constant (22). Similar changes were found in a murine model of cigarette smoke-induced emphysema (22). Although these reported findings support our general conclusion that changes in the ILC compartment are a feature of chronic lung inflammation, in detail, they contradict our present results showing total ILC numbers to be increased and the ILC1 proportion to be decreased in EM patients compared to LDs, possibly due to differences in identification strategies for ILCs and patient cohorts. Yet, in a smaller cohort of COPD and control patients, the authors found no increase in the proportion of ILC1s, but instead reported the ILC3 population to be increased in COPD compared to control patients (70.9% vs. 57.2% in COPD and control, respectively, sum of percentages for NCR- and NCR^+^ ILC3s) (23). Of note, the proportion of ILC3s in the control tissues in both referenced studies (22, 23) is substantially higher than in our present data set. In the studies referenced above, control subjects underwent lobectomy for solitary pulmonary tumors and included patients with a history of smoking. In our hands, preliminary analyses of lung tissue from patients undergoing surgery for bronchial adenocarcinoma, even though healthy tissue was resected outside the tumor area, also revealed an elevated proportion of ILC3s in the tissue compared to LDs without pulmonary malignancy (Michelle Paulsen, data not shown) and led us to refrain from using such tissue as a comparator. Scientifically, these observations suggest that even before lung failure and in less advanced COPD, smoking-induced damage may cause expansion of the ILC3 population and that overall, an enlarged ILC3 fraction is a sign of chronic inflammation in lung tissue.

Intriguingly, we find that besides type 3 ILCs, at least in lung-draining LNs from FI and EM patients, type 1 ILCs contribute to IL-17A production. Based on our flow cytometric analysis, there is little indication that the IL-17A-rich environment in endstage lungs is driven by enhanced cytokine production by ILCs on the per-cell level. Our co-expression analysis suggests that in the lung and LN tissues, ILCs may to some degree co-produce cytokines normally characterizing distinctly polarized immune responses. Furthermore, in both lung and LNs from all three endstage lung disease entities, we detect albeit small populations of ckit^+^ ILC2s, which have been reported can become producers of IL-17A and IL-22 (17, 18). Together, these findings support previous studies suggesting that ILCs may plastically adapt their tissue phenotype in response to cues from the chronic inflammatory environment in the lung. For example, under the influence of IL-12 and IL-18 released during murine influenza A infection, and also when treated with these cytokines *in vitro*, ILC2s have been shown to acquire ILC1-like properties (24). In that line, ILC2 function and IL-33-responsiveness can also be altered by cigarette smoke (25) and in nasal polyps from CF patients, ILC2s have been suggested to trans-differentiate into ILC3s under the combined impact of the inflammatory microenvironment, driven by IL-1β, IL-23, and TGF-β (26). Altogether, such findings suggest that ILC composition and phenotype are strongly dependent on the tissue context and that sustained changes in the composition may be indicative of chronic inflammation. Our data extend this concept to human endstage lung disease, suggesting that modulation of the processes involved in such plastic changes may represent a possible approach to therapeutically address lung failure.

In our present study, a change in the ILC composition was detectable not only in the lungs of CF patients at the stage of lung failure, but already in the PB of clinically stable patients eligible for treatment with ETI compared to healthy controls. Of interest, the difference observed in the PB of these MODULATE-CF participants compared to healthy controls was not towards ILC3-dominance but instead towards a more prominent ILC2 fraction at the expense of the ILC3 population. In contrast, a prior study has identified a reduction in the frequency of CCR6-expression ILC2s in the PB of CF patients, which correlates with decline in lung function (27). Supported by *in vitro* migration assays towards the CCR6 ligand CCL20 and transfer of human CF ILC2s into mice, the study suggests that CCR6^+^ ILC2s extravasate into lung tissue, where they enhance lung inflammation and reduce lung function (27). Our direct analysis of endstage CF lung tissue revealed an enlarged proportion of ILC3s rather than local ILC2 accumulation; however, a direct comparison with the above described findings is not feasible since our analyses did not include CCR6 expression. Analyzing CCR6-expressing ILCs in PB and lung tissue from the same patients, which was not technically feasible in our cohort, would provide more pertinent insight into the connection between ILC2 and ILC3 subpopulations in PB and lung. Further studies have reported yet other changes in the PB ILC compartment in CF. A conference abstract reports increases in the proportion of ILC1s in PB and broncho-alveolar lavage of CF patients compared to healthy controls, accompanied by a reduction in ILC3s, and once again links the ILC2 fraction with lung function (28). In COPD patients, a previous study found the proportion of PB ILC1s to be increased at the expense ILC2s in a manner that was directly associated with ppFEV_1_ and exacerbation frequency (24). The differences we observed between lung and PB ILC composition might reflect differences in disease stage, as the participants of the MODULATE-CF study show largely preserved lung function even prior to the initiation of therapy, which is evidently not the case in the endstage patients. Unfortunately, for ethical reasons, we do not have access to lung function data of the lung transplant recipients in our study to assess a potential covariation of lung function and ILC2s in lung tissue. However, due to the endstage nature of the participants’ lung disease, it seems reasonable to assume ppFEV1 values <40% for all participants, thereby rendering analyses of associations between lung function and ILC numbers challenging to impossible in our study population.

A simple view of the connection between PB ILC compartment und lung would be that PB ILC extravasation into the lung is the dominant migration route and entry of ILCs from PB into lung would be reflected in corresponding changes in both compartments. However, a wealth of evidence suggests that ILC compartments in PB and non-lymphoid tissue are determined by ILC plasticity and more complex ILC migration routes, with some of these previous findings also holding possible explanations for the differences between the PB and lung composition of ILCs we observed in our cohorts.

Concerning migration routes, ILCs have been referred to as tissue-resident cells for a long time. Indeed, some evidence from parabiosis experiments indicates that at least the ILC2 compartment in the lung is largely unaffected by “outside” events, even during local inflammation and infection (6). Instead, ILCs remaining in the tissue following prior challenges may serve to establish local immunological “memory” (29). However, beyond the fact that mature ILCs are detectable in the PB and LNs, there is also vast evidence of ILC recirculation and exchange between organs (27, 30–34). In particular, one study has provided evidence of ILC2s leaving the lung during allergic inflammation and entering the liver to promote inflammation (35). Comparable mechanisms may be envisioned in chronic inflammatory lung disease and suggest that alterations in the PB ILC compartment might not be directly proportional to the de- or increase of specific ILC subpopulations in the lung. More direct analysis of ILC migration routes, e.g. using direct cell tracking in mouse models (36, 37), as has been used to characterize skin ILC migration (38), appears a promising approach to advance our understanding about the relationship between the PB, lung, and LN ILC compartments in chronic lung disease, but is not feasible in humans.

With respect to plasticity, ckit^+^ ILC2s, which were detectable in MODULATE-CF PB samples but hardly in endstage lung tissue samples, have been reported to be able to acquire ILC3-like features and produce IL-17A (17, 18). One might envision a scenario in which the ckit^+^ ILC2 PB subset, which is enlarged in MODULATE-CF PB vs. HD PB, feeds the ILC3 compartment in the CF lung, with ckit^+^ ILC2s entering the lung and ILC2-to-ILC3 transition occurring rather immediately upon entry into the tissue under the influence of the chronically inflamed environment. Such a process may explain the discrepancy between the frequency of ckit^+^ ILCs detected in PBMCs vs. lung and LN tissues. Yet, our data can only partially support this hypothesis because disease progression and lung diseases status between MODULATE-CF participants and lung transplant recipients differ dramatically. Supportive of this scenario, in nasal polyps from CF patients, the ILC3 population was reported to be increased compared to non-CF patients with nasal polyps suffering from chronic rhino-sinusitis (26) and the authors suggested that ILC3s may have arisen from ILC2 through trans-differentiation triggered by the inflammatory microenvironment (39). Similarly, ILC2-to-ILC3-trans-differentiation, with ckit^+^ ILC2s being an intermediate state in-between ckit- ILC2s and ILC3s, may occur in endstage lungs. The detection of NCR^+^ ILC3s in endstage lungs but not PB may also be a result of ILC plasticity, with NCR- ILC3s acquiring the expression of natural cytotoxicity receptors NKp44 upon lung entry or with PB-derived NCR^+^ ILC3s expanding more efficiently in the lung than NCR- ILC3s. Altogether, additional studies are necessary to understand if the PB ILC compartment mirrors the situation in the lung or whether the ILC composition in these two compartments is driven by independent mechanisms.

In our recent study analyzing peripherally detectable inflammatory markers in the MODULATE-CF cohort (8), we have shown that PB neutrophil counts, C-reactive protein, serum G-CSF, and serum IL-6 are reduced strongly after initiation of a disease-modifying therapy with CFTR modulators and that these reductions correlate with improvements of lung function. However, inflammation levels did not attain healthy control levels for all parameters and in the sub-group of patients with increased inflammation prior to initiation of therapy, levels of systemic inflammation remain increased under treatment (8). The failure of the PB ILC composition to revert to a healthy state under ETI might therefore reflect residual inflammation not sufficiently targeted by this specific disease-modifying therapy. As such, our results advocate further exploration of ILCs and their secreted cytokines as potential targets to limit the deleterious consequences of an IL-17A-rich micro-environment responsible for inflammation and tissue destruction.

Altogether, we show that endstage lung disease and chronic lung inflammation are accompanied by sustained changes in the ILC compartment, favoring ILC3s and pro-inflammatory cytokine production in the lung. To our knowledge, our present study is the first to report on the ILC composition in CF lung tissue. Limitations of our study include the availability of tissue samples, reducing statistical power and preventing *a priori* sample size calculation, as well as the high inter-individual variability in disease trajectories and disease progression at the time of transplantation, which is reflected in the heterogeneity of our samples. This limitation must be emphasized particularly with respect to our data on immunofluorescence microscopic and chip cytometric analyses, which are based on small sample sizes and comparisons between only a few individual patients and must therefore be interpreted with caution. Also, age differences between our lung transplant recipients and MODULATE-CF participants as well as HDs and MODULATE-CF participants, must be taken into account. While we tried to assess the impact of age differences between HDs and MODULATE-CF participants through additional analysis of age-matched sub-cohorts, the age difference between lung transplant recipients and MODULATE-CF participants remains a caveat. Most certainly, COVID-19 mitigation measures with social distancing and reduced frequencies of seasonal respiratory infection (40) must also be kept in mind as a confounding factor in the MODULATE-CF cohort as well as in lung and LN samples obtained later than 2019, directly or indirectly impacting on the ILC compartment. In summary, however, based on our findings, the lung tissue ILC compartment may be considered a sensor reflecting the inflammatory state of the tissue and perpetuating, in case of endstage lung disease, a uniform “endstage” pattern of inflammation characterized by ILC3/IL-17 dominance. Our data on systemic ILC composition in more mildly affected CF patients furthermore suggest that such changes in the ILC compartment are challenging to target even with disease-modifying drugs and therefore advocate further studies to identify the mechanisms underlying these changes in ILC subpopulations as a potential target for anti-inflammtory approaches.

## Data Availability

The raw data supporting the conclusions of this article will be made available by the authors, without undue reservation.
