# Supplementary material for: Type 3 innate lymphoid cells dominate the ILC compartment in endstage lung disease
Source: Front Immunol. 2026 Jun 10;17:1716115. doi: 10.3389/fimmu.2026.1716115 (PMC13292765; doi:10.3389/fimmu.2026.1716115)
Supplement: Supplementary file 1 [file SupplementaryFile1.docx]

**SUPPLEMENTARY METHODS**

**Flow cytometric identification strategy for ILCs.**

Total ILCs were addressed as live CD45^+^CD127^+^ cells lacking the lineage (LIN) markers CD11c, CD19, CD14, CD4, CD34, CD3, and CD94 (Supplementary Figure 1A and [1]). There has been some controversy in the past on whether the frequently used marker CD161 constitutes a pan-ILC marker [2-6]. Because in the tissues analyzed, we found CD161 to be expressed preferentially on type 2 ILCs (Supplementary Figure 1B), we did not include this marker for the identification of total ILCs.

**Age-matching of HD and MODULATE-CF cohorts.**

For age-matching, patients from the HD and MODULATE-CF cohorts from whose PB an ILC subset composition analysis had been performed were sorted according to age and patients were consecutively excluded from the cohorts, starting with the youngest patients in the MODULATE-CF cohort and the oldest patients in the HD cohort, until mean and median age in the cohorts were approximately comparable. As a result of this, 29 of 39 patients were excluded from the MODULATE-CF cohort and 1 of 16 patients was excluded from the HD cohort. The age matched comparison was hence performed between 10 MODULATE-CF participants and 15 HDs as illustrated in the following figure.


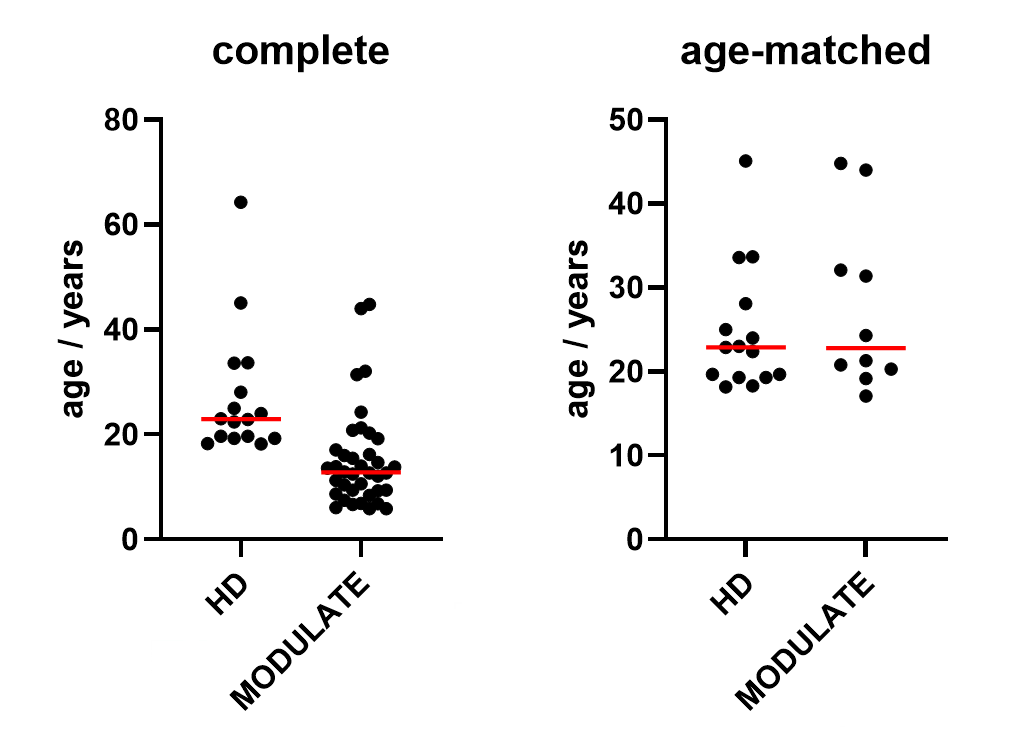


***Figure Age-matched ILC subset composition:*** *Analyzed groups of HD and MODULATE-CF participants with ILC subset composition analysis are shown before (“complete”, left panel) and following age-matching (“age-matched”, right panel). One dot per patient; horizontal line indicates the median.*

**Supplementary Figures**

**
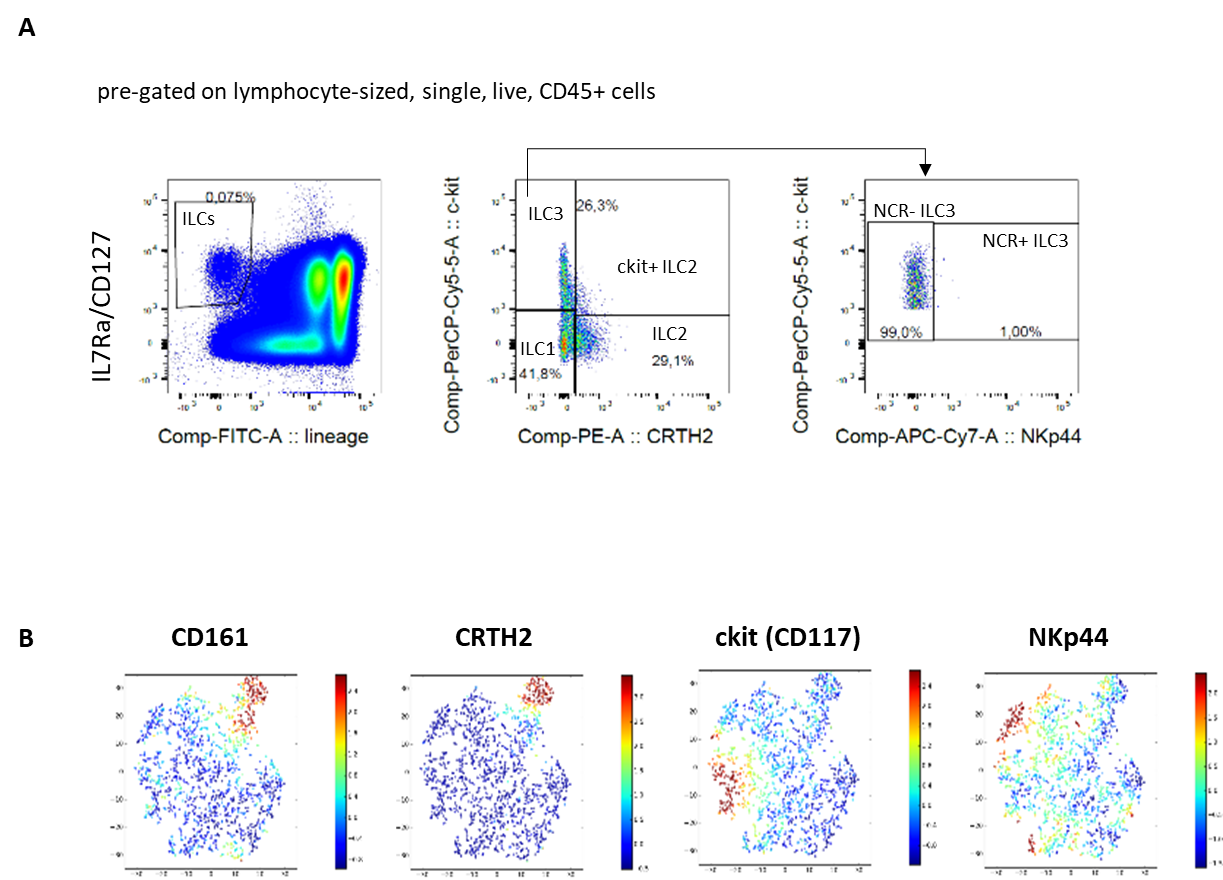
**

**Supplementary Figure 1. Strategies to address helper ILC subpopulations.**

A. Strategy to identify ILCs and ILC subtypes using flow cytometry. Among live lymphocyte-sized single cells, CD45+ cells are selected (not depicted), followed by gating on IL7Ra/CD127-positive and Lineage (CD11c, CD19, CD14, CD4, CD34, CD3, and CD94)-negative (Lin-) cells. The CD127+Lin- population is regarded as the pan-ILC population. Within the ILC population, using CRTH2 and ckit/CD117, we distinguish ILC1 (CRTH2-ckit-), ckit- ILC2 (ckit-CRTH2+), ckit+ ILC2 (ckit+CRTH2+) and ILC3s (CRTH2-ckit+). ILC3s are subdivided on the basis of NKp44 into natural cytotoxicity receptor (NCR)-negative (NKp44-) ILC3s and NCR+ (NKp44+) ILC3.

B. Preferential expression of CD161 on ILC2s. ILC subtypes ILC1, ILC2, and NCR+/- ILC3s in lung and lymph node tissues from CF, EM, FI, and LD were analyzed for expression of CD161. tSNE-Plots show expression of indicated markers in CD127+Lin- population (color-coded, see legend). Clustering on the basis of flow cytometric expression values was performed with Cell Accense. CD161 expression was consistently higher on ILC2s, as defined by CRTH2 expression, in all conditions. CD161 was therefore excluded from the group of markers used to identify pan-ILCs.

**Supplementary Table 1. Lung and Lymph node tissue samples**

| **Disease entity** | **Lung-ID** | **analysis method** | **LN-ID** | **analysis method** |
| --- | --- | --- | --- | --- |
| CF | G174 | ILC composition | LK174 | ILC composition |
| CF |  |  | LK176 | ILC composition |
| CF | G183 | ILC composition | LK183 | ILC composition |
| CF |  |  | LK187 | ILC composition |
| CF | G188 | ILC composition | LK188 | ILC composition |
| CF | G202 | ILC composition | LK202 | ILC composition |
| CF |  |  | LK206 | ILC composition; cytokine production |
| CF | G211 | ILC composition |  |  |
| CF | G236 | ILC composition | LK236 | ILC composition |
| CF | G246 | ILC composition | LK246 | ILC composition |
| CF | G257 | Histology |  |  |
| CF | G262 | Histology | LK262 | Histology |
| CF | G266 | ILC composition |  |  |
| CF | G271 | cytokine production | LK271 | cytokine production |
| CF | G289 | ILC composition | LK289 | ILC composition |
| CF | G294 | ILC composition | LK294 | ILC composition |
| CF | G306 | cytokine production |  |  |
| CF | G342 | cytokine production | LK342 | cytokine production |
| CF | G349 | cytokine production | LK349 | cytokine production |
| CF | G361 | cytokine production | LK361 | cytokine production |
| CF | G388 | cytokine production | LK388 | cytokine production |
| CF | G433 | chip cytometry | LK433 | chip cytometry |
| CF | G468 | cytokine production |  |  |
| CF | G523 | cytokine production | LK523 | cytokine production |
| CF | G533 | ILC composition | LK533 | ILC composition |
| EM | G168 | ILC composition | LK168 | ILC composition |
| EM | G172 | ILC composition | LK172 | ILC composition |
| EM |  |  | LK190 | ILC composition |
| EM |  |  | LK197 | ILC composition |
| EM | G205 | ILC composition | LK205 | ILC composition |
| EM | G208 | ILC composition | LK208 | ILC composition |
| EM | G219 | ILC composition | LK219 | ILC composition |
| EM |  |  | LK244 | ILC composition |
| EM |  |  | LK245 | ILC composition |
| EM | G255 | ILC composition |  |  |
| EM | G264 | ILC composition |  |  |
| EM | G273 | cytokine production; Histology | LK273 | cytokine production; Histology |
| EM | G274 | Histology |  |  |
| EM | G275 | ILC composition | LK275 | ILC composition |
| EM | G277 | ILC composition | LK277 | ILC composition |
| EM | G285 | cytokine production | LK285 | cytokine production |
| EM | G297 | ILC composition | LK297 | ILC composition |
| EM | G298 | ILC composition | LK298 | ILC composition |
| EM | G300 | cytokine production | LK300 | cytokine production |
| EM | G304 | cytokine production | LK304 | cytokine production |
| EM | G307 | ILC composition | LK307 | ILC composition |
| EM | G308 | cytokine production |  |  |
| EM | G319 | cytokine production | LK319 | cytokine production |
| EM | G331 | cytokine production | LK331 | cytokine production |
| EM | G334 | cytokine production | LK334 | cytokine production |
| EM | G336 | cytokine production |  |  |
| EM | G338 | cytokine production | LK338 | cytokine production |
| EM | G340 | cytokine production | LK340 | cytokine production |
| EM |  |  | LK355 | cytokines in subsets |
| EM |  |  | LK357 | cytokines in subsets |
| EM | G362 | cytokines in subsets | LK362 | cytokines in subsets |
| EM | G366 | cytokines in subsets |  |  |
| EM | G406 | chip cytometry |  |  |
| EM |  |  | LK418 | chip cytometry |
| EM |  |  | LK435 | chip cytometry |
| EM |  |  | LK551 | cytokines in subsets |
| EM | G552 | cytokines in subsets | LK552 | cytokines in subsets |
| EM | G554 | cytokines in subsets | LK554 | cytokines in subsets |
| EM |  |  | LK556 | cytokines in subsets |
| EM | G556 | cytokines in subsets |  |  |
| EM | G557 | cytokines in subsets | LK557 | cytokines in subsets |
| EM |  |  | LK559 | cytokines in subsets |
| EM | G561 | cytokines in subsets | LK561 | cytokines in subsets |
| EM | G562 | cytokines in subsets | LK562 | cytokines in subsets |
| EM |  |  | LK568 | cytokines in subsets |
| EM | G570 | cytokines in subsets | LK570 | cytokines in subsets |
| EM | G572 | cytokines in subsets | LK572 | cytokines in subsets |
| EM | G577 | cytokines in subsets | LK577 | cytokines in subsets |
| EM | G581 | cytokines in subsets | LK581 | cytokines in subsets |
| FI | G166 | ILC composition | LK166 | ILC composition |
| FI | G170 | ILC composition | LK170 | ILC composition |
| FI |  |  | LK178 | ILC composition |
| FI | G185 | ILC composition | LK185 | ILC composition |
| FI |  |  | LK199 | ILC composition |
| FI | G200 | ILC composition |  |  |
| FI | G223 | ILC composition | LK223 | ILC composition |
| FI | G226 | ILC composition | LK226 | ILC composition |
| FI | G229 | ILC composition |  |  |
| FI | G231 | ILC composition | LK231 | ILC composition |
| FI | G238 | ILC composition | LK238 | ILC composition |
| FI |  |  | LK240 | ILC composition |
| FI | G253 | ILC composition | LK253 | ILC composition |
| FI | G268 | Histology | LK268 | Histology |
| FI | G279 | cytokine production; Histology | LK279 | cytokine production; Histology |
| FI | G281 | cytokine production | LK281 | cytokine production |
| FI | G287 | cytokine production | LK287 | cytokine production |
| FI | G310 | cytokine production |  |  |
| FI | G312 | cytokine production |  |  |
| FI | G316 | cytokine production | LK316 | cytokine production |
| FI | G317 | cytokine production | LK317 | cytokine production |
| FI |  |  | LK320 | cytokine production |
| FI | G321 | cytokine production | LK321 | cytokine production |
| FI | G323 | cytokine production | LK323 | cytokine production |
| FI | G330 | cytokine production | LK330 | cytokine production |
| FI |  |  | LK332 | cytokine production |
| FI | G358 | cytokines in subsets | LK358 | cytokines in subsets |
| FI | G364 | cytokines in subsets |  |  |
| FI | G404 | ILC composition | LK404 | ILC composition |
| FI | G423 | chip cytometry | LK423 | chip cytometry |
| FI | G439 | chip cytometry | LK439 | chip cytometry |
| FI | G542 | cytokines in subsets | LK542 | cytokines in subsets |
| FI | G564 | cytokines in subsets |  |  |
| LD |  |  | LK167 | ILC composition |
| LD |  |  | LK169 | ILC composition |
| LD |  |  | LK171 | ILC composition |
| LD |  |  | LK173 | ILC composition |
| LD |  |  | LK175 | ILC composition |
| LD |  |  | LK177 | ILC composition |
| LD |  |  | LK184 | ILC composition |
| LD |  |  | LK186 | ILC composition |
| LD |  |  | LK189 | ILC composition |
| LD |  |  | LK191 | ILC composition |
| LD |  |  | LK201 | ILC composition |
| LD |  |  | LK203 | ILC composition |
| LD |  |  | LK204 | ILC composition |
| LD |  |  | LK207 | ILC composition |
| LD |  |  | LK210 | ILC composition |
| LD |  |  | LK220 | ILC composition |
| LD |  |  | LK224 | ILC composition |
| LD |  |  | LK232 | ILC composition |
| LD |  |  | LK241 | ILC composition |
| LD |  |  | LK243 | ILC composition |
| LD |  |  | LK247 | ILC composition |
| LD |  |  | LK254 | ILC composition |
| LD |  |  | LK261 | Histology |
| LD |  |  | LK276 | ILC composition |
| LD |  |  | LK280 | cytokine production |
| LD |  |  | LK282 | cytokine production |
| LD | G288 | ILC composition; Histology | LK288 | cytokine production; Histology |
| LD |  |  | LK305 | cytokine production |
| LD |  |  | LK313 | cytokine production |
| LD |  |  | LK318 | cytokine production |
| LD |  |  | LK322 | cytokine production |
| LD |  |  | LK324 | cytokine production |
| LD |  |  | LK333 | cytokine production |
| LD |  |  | LK335 | cytokine production |
| LD |  |  | LK337 | cytokine production |
| LD |  |  | LK339 | cytokine production |
| LD |  |  | LK341 | cytokine production |
| LD | G348 | ILC composition |  |  |
| LD |  |  | LK351 | cytokine production |
| LD |  |  | LK356 | cytokines in subsets |
| LD |  |  | LK359 | cytokines in subsets |
| LD |  |  | LK363 | cytokines in subsets |
| LD | G371 | ILC composition |  |  |
| LD |  |  | LK389 | cytokine production |
| LD | G420 | chip cytometry |  |  |
| LD |  |  | LK434 | chip cytometry |
| LD | G446 | ILC composition |  |  |
| LD | G467 | ILC composition |  |  |
| LD |  |  | LK469 | cytokine production |
| LD | G478 | ILC composition |  |  |
| LD | G507 | ILC composition |  |  |
| LD | G518 | ILC composition | LK518 | ILC composition |
| LD |  |  | LK534 | ILC composition |
| LD |  |  | LK560 | cytokines in subsets |
| LD |  |  | LK563 | cytokines in subsets |
| LD |  |  | LK565 | cytokines in subsets |
| LD |  |  | LK571 | cytokines in subsets |
| LD |  |  | LK578 | cytokines in subsets |

*IDs Gxxx refer to lung tissue; IDs LKxxx refer to LN tissue; tissues with identical 3-digit number were obtained from the same patient; CF, cystic fibrosis; EM, COPD/ emphysema; FI, lung fibrosis; LD, lung donor.*

**Supplementary Table 2. Healthy donors and MODULATE-CF participants**

| **Patient-ID** | **Sex** | **Age (years) median (IQR)** | **ppFEV1 (MODULATE-CF baseline)** |
| --- | --- | --- | --- |
| Healthy Donors | | | |
| n=16 | f: 12; m: 4 | 23.0 (19.6-29.5) | n.d. |
| Healthy Donor_06 | f | 18.2 |  |
| Healthy Donor_05 | f | 18.3 |  |
| Healthy Donor_02 | f | 19.3 |  |
| Healthy Donor_04 | f | 19.3 |  |
| Healthy Donor_12 | f | 19.7 |  |
| Healthy Donor_11 | f | 22.9 |  |
| Healthy Donor_08 | f | 23.0 |  |
| Healthy Donor_07 | f | 24.0 |  |
| Healthy Donor_01 | f | 25.0 |  |
| Healthy Donor_09 | f | 28.1 |  |
| Healthy Donor_15 | f | 45.1 |  |
| Healthy Donor_14 | f | 64.3 |  |
| Healthy Donor_03 | m | 19.7 |  |
| Healthy Donor_10 | m | 22.4 |  |
| Healthy Donor_16 | m | 33.6 |  |
| Healthy Donor_13 | m | 33.7 |  |
| Modulate-CF participants | | | |
| n=48 | f:25; m:23 | 13.9 (9.4-19.5) | 88.0 (74.5-100.5) |
| Modulate-CF_34 | f | 6.1 | 115 |
| Modulate-CF_33 | f | 7.5 | 88 |
| Modulate-CF_36 | f | 8.4 | 87 |
| Modulate-CF_41 | f | 9.3 | 88 |
| Modulate-CF_40 | f | 9.3 | 96 |
| Modulate-CF_42 | f | 11.3 | 94 |
| Modulate-CF_19 | f | 12.1 | 63 |
| Modulate-CF_17 | f | 12.5 | 53 |
| Modulate-CF_13 | f | 12.7 | 101 |
| Modulate-CF_06 | f | 13.6 | 107 |
| Modulate-CF_12 | f | 13.7 | 110 |
| Modulate-CF_10 | f | 13.8 | 129 |
| Modulate-CF_09 | f | 14.0 | 85 |
| Modulate-CF_31 | f | 14.2 | 74 |
| Modulate-CF_02 | f | 14.7 | 104 |
| Modulate-CF_21 | f | 15.4 | 69 |
| Modulate-CF_28 | f | 15.5 | 113 |
| Modulate-CF_01 | f | 16.0 | 93 |
| Modulate-CF_11 | f | 17.2 | 71 |
| Modulate-CF_15 | f | 20.8 | 48 |
| Modulate-CF_04 | f | 21.3 | 53 |
| Modulate-CF_22 | f | 24.3 | 94 |
| Modulate-CF_05 | f | 29.0 | 66 |
| Modulate-CF_25 | f | 41.2 | 94 |
| Modulate-CF_38 | f | 8.7 | NA |
| Modulate-CF_48 | m | 5.9 | 98 |
| Modulate-CF_47 | m | 5.9 | NA |
| Modulate-CF_35 | m | 6.7 | 92 |
| Modulate-CF_45 | m | 6.8 | 108 |
| Modulate-CF_37 | m | 6.9 | 101 |
| Modulate-CF_39 | m | 9.4 | 102 |
| Modulate-CF_44 | m | 9.4 | 102 |
| Modulate-CF_46 | m | 10.4 | 88 |
| Modulate-CF_43 | m | 10.6 | 82 |
| Modulate-CF_03 | m | 12.7 | 81 |
| Modulate-CF_08 | m | 12.9 | 72 |
| Modulate-CF_26 | m | 13.9 | 95 |
| Modulate-CF_27 | m | 16.2 | 87 |
| Modulate-CF_32 | m | 16.8 | 73 |
| Modulate-CF_07 | m | 17.1 | 84 |
| Modulate-CF_30 | m | 19.2 | 99 |
| Modulate-CF_24 | m | 20.3 | 101 |
| Modulate-CF_23 | m | 20.7 | 80 |
| Modulate-CF_29 | m | 22.1 | 96 |
| Modulate-CF_16 | m | 31.4 | 64 |
| Modulate-CF_20 | m | 32.1 | 76 |
| Modulate-CF_14 | m | 44.0 | 82 |
| Modulate-CF_18 | m | 44.8 | 60 |

**Supplementary References**

1. Cossarizza A, Chang HD, Radbruch A, Abrignani S, Addo R, Akdis M, Andra I, Andreata F, Annunziato F, Arranz E, Bacher P, Bari S, Barnaba V, Barros-Martins J, Baumjohann D, Beccaria CG, Bernardo D, Boardman DA, Borger J, Bottcher C, Brockmann L, Burns M, Busch DH, Cameron G, Cammarata I, Cassotta A, Chang Y, Chirdo FG, Christakou E, Cicin-Sain L, Cook L, Corbett AJ, Cornelis R, Cosmi L, Davey MS, De Biasi S, De Simone G, Del Zotto G, Delacher M, Di Rosa F, Di Santo J, Diefenbach A, Dong J, Dorner T, Dress RJ, Dutertre CA, Eckle SBG, Eede P, Evrard M, Falk CS, Feuerer M, Fillatreau S, Fiz-Lopez A, Follo M, Foulds GA, Frobel J, Gagliani N, Galletti G, Gangaev A, Garbi N, Garrote JA, Geginat J, Gherardin NA, Gibellini L, Ginhoux F, Godfrey DI, Gruarin P, Haftmann C, Hansmann L, Harpur CM, Hayday AC, Heine G, Hernandez DC, Herrmann M, Hoelsken O, Huang Q, Huber S, Huber JE, Huehn J, Hundemer M, Hwang WYK, Iannacone M, Ivison SM, Jack HM, Jani PK, Keller B, Kessler N, Ketelaars S, Knop L, Knopf J, Koay HF, Kobow K, Kriegsmann K, Kristyanto H, Krueger A, Kuehne JF, Kunze-Schumacher H, Kvistborg P, Kwok I, Latorre D, Lenz D, Levings MK, Lino AC, Liotta F, Long HM, Lugli E, MacDonald KN, Maggi L, Maini MK, Mair F, Manta C, Manz RA, Mashreghi MF, Mazzoni A, McCluskey J, Mei HE, Melchers F, Melzer S, Mielenz D, Monin L, Moretta L, Multhoff G, Munoz LE, Munoz-Ruiz M, Muscate F, Natalini A, Neumann K, Ng LG, Niedobitek A, Niemz J, Almeida LN, Notarbartolo S, Ostendorf L, Pallett LJ, Patel AA, Percin GI, Peruzzi G, Pinti M, Pockley AG, Pracht K, Prinz I, Pujol-Autonell I, Pulvirenti N, Quatrini L, Quinn KM, Radbruch H, Rhys H, Rodrigo MB, Romagnani C, Saggau C, Sakaguchi S, Sallusto F, Sanderink L, Sandrock I, Schauer C, Scheffold A, Scherer HU, Schiemann M, Schildberg FA, Schober K, Schoen J, Schuh W, Schuler T, Schulz AR, Schulz S, Schulze J, Simonetti S, Singh J, Sitnik KM, Stark R, Starossom S, Stehle C, Szelinski F, Tan L, Tarnok A, Tornack J, Tree TIM, van Beek JJP, van de Veen W, van Gisbergen K, Vasco C, Verheyden NA, von Borstel A, Ward-Hartstonge KA, Warnatz K, Waskow C, Wiedemann A, Wilharm A, Wing J, Wirz O, Wittner J, Yang JHM, Yang J. Guidelines for the use of flow cytometry and cell sorting in immunological studies (third edition). *Eur J Immunol* 2021: 51(12): 2708-3145.

2. Trabanelli S, Gomez-Cadena A, Salome B, Michaud K, Mavilio D, Landis BN, Jandus P, Jandus C. Human innate lymphoid cells (ILCs): Toward a uniform immune-phenotyping. *Cytometry B Clin Cytom* 2018: 94(3): 392-399.

3. Lanier LL. Plastic fantastic innate lymphoid cells. *J Exp Med* 2019: 216(8): 1726-1727.

4. Li N, van Unen V, Hollt T, Thompson A, van Bergen J, Pezzotti N, Eisemann E, Vilanova A, Chuva de Sousa Lopes SM, Lelieveldt BPF, Koning F. Mass cytometry reveals innate lymphoid cell differentiation pathways in the human fetal intestine. *J Exp Med* 2018: 215(5): 1383-1396.

5. Simoni Y, Fehlings M, Kloverpris HN, McGovern N, Koo SL, Loh CY, Lim S, Kurioka A, Fergusson JR, Tang CL, Kam MH, Dennis K, Lim TKH, Fui ACY, Hoong CW, Chan JKY, Curotto de Lafaille M, Narayanan S, Baig S, Shabeer M, Toh SES, Tan HKK, Anicete R, Tan EH, Takano A, Klenerman P, Leslie A, Tan DSW, Tan IB, Ginhoux F, Newell EW. Human Innate Lymphoid Cell Subsets Possess Tissue-Type Based Heterogeneity in Phenotype and Frequency. *Immunity* 2018: 48(5): 1060.

6. Mjosberg JM, Trifari S, Crellin NK, Peters CP, van Drunen CM, Piet B, Fokkens WJ, Cupedo T, Spits H. Human IL-25- and IL-33-responsive type 2 innate lymphoid cells are defined by expression of CRTH2 and CD161. *Nat Immunol* 2011: 12(11): 1055-1062.
